# Attitude and Awareness of Dental Students Regarding Malocclusion and Orthodontic Treatment

**DOI:** 10.7759/cureus.77224

**Published:** 2025-01-10

**Authors:** Waseem Khan, Parag Gangurde, Alok Ranjan, Harsh Mishra, Hitesh Sawant

**Affiliations:** 1 Department of Orthodontics, Bharati Vidyapeeth (Deemed to be University) Dental College and Hospital, Navi Mumbai, IND

**Keywords:** dental awareness, dental students' awareness, malocclusion, orthodontic awareness, orthodontic treatment

## Abstract

Objective: The purpose of this research is to assess dentistry students' knowledge and perspective on malocclusion and orthodontic therapy.

Methods: The research group consisted of 240 undergraduate dentistry students in all. One hundred and sixty Bachelor of Dental Surgery (BDS) students from each of the four years of study were chosen for the study. Students were asked to fill out a standardized questionnaire to gather data for this research. To check for statistical differences, an analysis of variance was conducted between the groups. Using a chi-squared test with a significance threshold of p < 0.05, categorical variables were examined.

Results: The vast majority of participants (98%) valued healthy, well-spaced teeth and were self-aware when it came to their dental esthetics. As far as dental esthetics, 70% of participants were happy. Nearly 89% of those surveyed agreed having straight teeth would make them look better. When asked about their feelings about orthodontic treatment, 12.5% gave it a very good response.

Conclusion: Findings indicate that participants understood the impact of their teeth on their appearance and the potential benefits of orthodontic treatment. People who were treated with orthodontic braces felt good about it. All four years of BDS students fall within the same age range (17-25); hence, there was no discernible difference in their perspectives on malocclusion and orthodontic treatment, as well as their level of understanding and attitude toward these topics.

## Introduction

Concerned with the diagnosis, treatment, and prevention of malocclusion and other dentofacial disorders, orthodontics is a subspecialty of dentistry. Any occlusion where the arches of the teeth do not meet in a normal connection or where there are abnormalities in the position of the teeth that extend beyond the typical boundaries is called malocclusion. Malocclusion can be caused by different factors, such as deleterious oral habits, supernumerary teeth, congenitally missing teeth, the shape of the dentition, and defects in the developmental position of teeth [1]. Malocclusion hurts periodontal health, causes temporomandibular joint problems, and also causes dental caries [2]. So, dental students need to have detailed knowledge regarding malocclusion.

In India, malocclusion is a neglected dental problem. More importance is given to problems related to dental caries and periodontium because of the pain experienced by patients due to them [3]. Orthodontic treatment is mostly carried out to improve the dental as well as facial appearance of the patient. Orthodontic treatment forms the most important part of esthetic dentistry.

On top of that, understanding how patients feel about malocclusion is becoming just as crucial in orthodontics. Esthetic enhancement and psychological considerations are the most prevalent drivers of orthodontic treatment decisions. It is crucial to establish the frequency of malocclusion in order to arrange orthodontic therapy. Being cognizant of something is the definition of awareness [4]. There are a number of stages to the decision-making process that an individual goes through while evaluating the esthetic pleasure of their own teeth, in order to learn how they perceive orthodontic treatment, how satisfied they are with their teeth, and how self-aware they are of their teeth [5].

In this study, the students were asked to fill out a questionnaire to evaluate their attitude and awareness regarding malocclusion and orthodontic treatment. The diagnosis of any dental abnormality is very important to render proper treatment to the patient. A critical evaluation of the complete set of dentition with associated structures is necessary for the proper diagnosis of any malocclusion at an early stage, which would help to deliver the best possible treatment to the patient.

Complete dental care delivered by dental professionals includes fulfilling the functional as well as esthetic requirements of the patient. In convincing the patient to orthodontic treatment, the most important part is played by the appearance of the dentist himself. The well-aligned teeth of the dentist will definitely have a good impact on the patient.

## Materials and methods

Over 240 dental students took part in this research project. The selection of participants was done at random. Students enrolled in the Bachelor of Dental Surgery (BDS) program were selected from each year to include 60 individuals. The information that was required for this research was gathered through the use of classroom observations as well as the presentation of a structured questionnaire. The questionnaire has been validated with a face value of one and Cronbach's alpha value of 0.8 with a check from subject experts.

The participants must meet the inclusion requirements, which include being enrolled as full-time undergraduate dental students at the institution that is conducting the study and having a successful academic record. Several characteristics are considered for the exclusion criteria. These include a history of craniofacial defects, substantial cognitive impairment, or previous orthodontic treatment. The following is a breakdown of the questions: There were seven questions related to dental awareness, four questions about self-satisfaction, and six questions about attitudes toward orthodontic treatment. For every question, there were three different choices available, and each one could be scored on a scale from one to three, with one being the best and two being the worst alternative. Both positive and negative responses are possible from the audience. Through the use of the questionnaire, the participant's comprehension of the repercussions of malocclusion, their perspective on orthodontic treatment, and their capacity to recognize the presence or absence of malocclusion were evaluated.

The questionnaire was distributed among students in the lecture hall, and they were asked to fill it out. The filled forms were collected back, and responses were recorded.

Scoring criteria

The three-point Likert scale was used for recording responses.

Statistical analysis

The sample size was calculated using the following formula:

n = Z2⋅P⋅(1 − P)/E2,

where Z = 1.96 (for a 95% confidence level), P = 0.5 (or your estimated proportion), and E = 0.05 (or your desired margin of error).

To conduct a statistical analysis of the data, the responses were first divided into three categories: positive responses, median answers, and unfavorable reactions. To evaluate the categorical variables, a chi-squared test was utilized. We have used a significance level of p < 0.05 in my analysis.

## Results

The 60 participants were selected from first-, second-, third-, and fourth-year BDS students (Table 1).

**Table 1:** Distribution of students according to year and sex n: number of students; %: percentage; BDS: Bachelor of Dental Surgery

Students	Sex		Total
	Males n(%)	Females n(%)	
1st-yr BDS	14 (23.33%)	46 (76.66%)	60
2nd-yr BDS	18 (30%)	42 (70%)	60
3rd-yr BDS	16 (26.66%)	44 (73.33%)	60
4th-yr BDS	17 (28.33%)	43 (71.66%)	60

While assessing the awareness of the subjects about their dental esthetics, it was found that 98% of the subjects were aware of their dental esthetics and were in favor of healthy and well-arranged teeth. Seventy percent of the sample was satisfied with their dental esthetics, 23% were not satisfied, and 7% of the subjects had a neutral attitude with a significant p-value (p = 0.05).

Eleven percent of the subjects reported difficulty speaking or chewing and facial muscle pain caused by tooth arrangement. About 79% of the subjects thought that their smile could be better if their teeth were well aligned (Table 2).

**Table 2:** Awareness of their dental esthetics Data is presented in the form of n(%) n: number of students; %: percentage; BDS: Bachelor of Dental Surgery

Questions	1st-yr BDS (n-60)	2nd-yr BDS (n-60)	3rd-yr BDS (n-60)	4th-yr BDS (n-60)	Statistical significance
Do you think healthy and well-arranged teeth are important for your appearance?					
Yes	58 (96%)	59 (98.3%)	58 (96%)	60 (100%)	p = 0.677
No	1 (1.6%)	1 (1.6%)	1 (1.6%)	0	
Don’t know	1 (1.6%)	0	1 (1.6%)	0	
Are you satisfied with your dental appearance?					
Yes	45 (75%)	41 (68.3%)	37 (61.6%)	46 (76.6%)	p = 0.005
No	7 (11.6%)	15 (25%)	21 (35%)	13 (21.6%)	
Don’t know	8 (13.3%)	4 (6.6%)	2 (33.3%)	1 (1.6%)	
How do you feel about the appearance of your teeth?					
Positive	37 (61.6%)	38 (63.3%)	38 (63.3%)	37 (61.6%)	p = 0.609
Negative	5 (8.3%)	7 (11.6%)	8 (13.3%)	11 (18.3%)	
Neutral	18 (30%)	15 (25%)	14 (23.3%)	12 (20%)	
Do you have any trouble while speaking or chewing or facial muscle pains caused by teeth arrangement?					
Yes	4 (6.6%)	5 (8.3%)	5 (8.3%)	12 (20%)	p = 0.056
No	56 (93.3%)	54 (90%)	52 (86.6%)	47 (78.3%)	
Don’t know	0	1 (1.6%)	3 (5%)	1 (1.6%)	
Do you think you should have orthodontic treatment?					
Yes	18 (30%)	17 (28.3%)	26 (43.3%)	24 (40%)	p = 0.009
No	29 (48.3%)	40 (66.6%)	28 (46.3%)	34 (56.6%)	
Don’t know	13 (21.6%)	3 (5%)	6 (10%)	2 (3.3%)	
Would you agree readily to orthodontic treatment if a dentist or parent suggested it?					
Yes	43 (71.6%)	52 (86.6%)	41 (68.3%)	43 (71.6%)	p = 0.106
No	12 (20%)	5 (8.3%)	13 (21.6%)	15 (25%)	
Don’t know	5 (8.3%)	3 (5%)	6 (10%)	2 (3.3%)	
Do you think your smile could be better if your teeth were better aligned?					
Yes	50 (83.3%)	50 (83.3%)	47 (78.3%)	44 (73.3%)	p = 0.520
No	6 (10%)	7 (11.6%)	9 (15%)	13 (21,6%)	
Don’t know	4 (6.6%)	3 (5%)	4 (6.6%)	3 (5%)	

While assessing the self-satisfaction of the subjects with their dental esthetics, it was found that about 77% of the samples were satisfied with the attractiveness of their teeth, and 15% of the samples reported a negative feeling toward their teeth. About 14% of subjects reported that they avoided smiling because of the appearance of their teeth, with a non-significant p-value (Table 3).

**Table 3:** Self-satisfaction with dental esthetics Data is presented in the form of n(%) n: number of students; %: percentage; BDS: Bachelor of Dental Surgery

Questions	1st-yr BDS (n-60)	2nd-yr BDS (n-60)	3rd-yr BDS (n-60)	4th-yr BDS (n-60)	Statistical significance
Have you found that other people have commented on the appearance of your teeth?					
Yes	23 (38.3%)	24 (40%)	21 (35%)	26 (43.3%)	p = 0.893
No	32 (53.3%)	33 (55%)	36 (60%)	29 (48.3%)	
Don’t know	5 (8.3%)	3 (5%)	3 (5%)	5 (8.3%)	
Have you found that other people have teased you on the appearance of your teeth?					
Yes	9 (15%)	9 (15%)	5 (8.3%)	15	p = 0.234
No	45 (75%)	46 (76.6%)	52 (86.6%)	42 (70%)	
Don’t know	6 (10%)	5 (8.3%)	3 (5%)	3 (5%)	
Do you try to avoid smiling because of the appearance of your teeth?					
Positive	9 (15%)	8 (13.3%)	10 (16.6%)	7 (11.6%)	p = 0.714
Negative	51 (85%)	52 (86.6%)	49 (81.6%)	53 (88.3%)	
Neutral	0	0	1 (1.6%)	0	
Do you ever cover your mouth because of your teeth?					
Yes	6 (10%)	7 (11.6%)	9 (15%)	9 (15%)	p = 0.514
No	54 (90%)	50 (83.3%)	49 (81.6%)	51 (85%)	
Don’t know	0	3 (5%)	2 (3.3%)	0	

There was a statistically significant difference between the two groups, with 28% reporting high levels of satisfaction and 61% expressing low levels (p = 0.528). Sixty-two percent of the samples had negative feelings about the way their teeth looked (Table 4).

**Table 4:** Attitude toward orthodontic treatment Data is presented in the form of n(%) n: number of students; %: percentage; BDS: Bachelor of Dental Surgery

Questions	1st-yr BDS (n-60)	2nd-yr BDS (n-60)	3rd-yr BDS (n-60)	4th-yr BDS (n-60)	Statistical significance
What kind of teeth do you have?					
Positive	39 (65%)	36 (60%)	31 (51.6%)	39 (65%)	p = 0.066
Negative	0	6 (10%)	8 (13.3%)	8 (13.3%)	
Neutral	21 (33.3%)	18 (30%)	21 (35%)	13 (21.6%)	
Are your teeth well aligned?					
Positive	38 (63.3%)	38 (63.3%)	28 (46.6%)	31 (51.6%)	p = 0.088
Negative	8 (13.3%)	14 (23.3%)	15 (25%)	19 (31.6%)	
Neutral	14 (23.3%)	8 (13.3%)	17 (28.3%)	10 (16.6%)	
How much do you like the way your teeth look?					
Positive	42 (70%)	38 (63.3%)	34 (56.6%)	39 (65%)	p = 0.167
Negative	2 (3.3%)	6 (10%)	8 (13.3%)	8 (13.3%)	
Neutral	16 (26.6%)	16 (26.6%)	18 (30%)	13 (21.6%)	
How much do your teeth affect the way your face looks?					
Positive	41 (68.3%)	30 (50%)	35 (58.3%)	36 (60%)	p = 0.578
Negative	6 (.310%)	8 (13.3%)	8 (13.3%)	9 (15%)	
Neutral	13 (21.6%)	22 (36.6%)	17 (2%)	15 (25%)	
Do you think your smile could be better if your teeth were better aligned?					
Positive	45 (75%)	48 (80%)	43 (73.8%)	41 (68.3%)	p = 0.110
Negative	4 (6.6%)	7 (7.7%)	9 (15%)	13 (21.6%)	
Neutral	11 (18.3%)	5 (8.3%)	8 (13.3%)	6 (10%)	

Twelve percent of the participants had a very good attitude about orthodontic treatment, whereas 74% had a very negative attitude.

## Discussion

To evaluate and treat malocclusions early in their practice, dental students should be knowledgeable about the condition and its symptoms. All age groups, from kids to adults, are becoming more cognizant of orthodontics as a field of dentistry. Dental students' perspectives on malocclusion and orthodontic treatment, as well as their understanding of the issues, are crucial. The desire to have a more attractive dentofacial profile is a major driving force for orthodontic treatment [6].

People who were unhappy with the way their teeth looked were open to the idea of orthodontic treatment. Those who reported high levels of esthetic satisfaction were also aware of the fact that their teeth looked great. The results show that the participants understood the esthetic value of properly spaced teeth. Even though they were dissatisfied with their appearance, the individuals were open to the idea of orthodontic treatment and would be willing to have braces if their dentist or parents suggested it. The difference in attitude and awareness among male and female subjects was evaluated in the study because of the huge difference in the sample size, i.e., the majority of the subjects were female. The gender gap in perception arises from the fact that women are more overtly depicted in media as having an ideal face shape [7]. Therefore, women place a higher value on changes in dental shape and structure, which impact the beauty or contour of the face, than men do [6]. People with malocclusion who did not bother to visit the orthodontic clinic were the least concerned about their teeth's appearance and were thus not eligible for orthodontic treatment due to their ignorance and carelessness. Participants in this research were self-aware when it came to their smile esthetics and were open to the idea of orthodontic treatment.

Kerosuo et al. [8] investigated orthodontic treatment, individuals' judgments of their teeth and dental appearance, and their subjective need for treatment in connection to demographic variables including the financing system, place of residence, age, gender, ethnicity, and socioeconomic position. Although the findings did not show any effect on the self-perceived need for treatment, the availability of free orthodontic therapy was likely to alter the treatment rate. In the present study, the subjects were of similar socioeconomic status, so their perception of dental esthetics was not affected by their socioeconomic status.

A similar study conducted by Helm et al. in 1985 [9] hypothesized that certain malocclusions, particularly noticeable space and occlusal abnormalities, would hurt self-concept and body image throughout life. Children and parents alike consider esthetically pleasing environments to be crucial to their mental and emotional health, according to research published in 2000 by Birkeland et al. [10]. In a study conducted by Bantele et al. [11], after careful consideration, they concluded that the index of orthodontic treatment need (IOTN) had great potential as a tool to enhance orthodontic referral education. It was feasible to teach the students within the time limits of a dentistry school curriculum by combining computer-generated and hands-on learning. A study was conducted by Kamath and Arun [12] to identify the variables impacting the demand for orthodontic treatment among Saveetha Dental College outpatients. The research found that the biggest reason individuals do not get orthodontic treatment is because they do not know about it. Few individuals in the research were motivated by their own perceptions about orthodontic treatment.

The influence of dental esthetics on self-esteem and social interactions is well documented. A study by Bos et al. revealed that individuals with perceived dental imperfections are more likely to exhibit lower self-confidence and higher social anxiety, indicating the psychosocial implications of dental esthetics [13]. This underscores the importance of educating dental students not only on the technical aspects of orthodontic treatment but also on its psychosocial benefits. Sociocultural perceptions play a significant role in the acceptance and pursuit of orthodontic treatment. Langlois et al. found that societal standards of beauty significantly affect individuals' satisfaction with their own dental esthetics and their likelihood of seeking orthodontic treatment [14]. This aspect suggests that dental education should include components that address societal influences on patient decisions, preparing future dentists to better understand and manage patient expectations.

Technological advancements in orthodontics, such as the development of clear aligners, have made treatments less conspicuous, thereby increasing their appeal among adults. A review by Weir highlights the impact of these innovations on patient choices and treatment accessibility [15]. Including these advancements in the dental curriculum can provide students with up-to-date knowledge and skills in modern orthodontic solutions. Economic factors are a significant barrier to accessing orthodontic treatment. McGrath and Bedi explored how socioeconomic status affects the availability and quality of orthodontic treatment among different demographics [16]. Understanding these economic barriers and discussing possible solutions, such as flexible payment plans and public health policies, are crucial for dental education. Effective educational strategies are crucial for enhancing the knowledge and skills of dental students in orthodontics. A study by Sipiyaruk et al. emphasized the importance of simulation and digital tools in dental education to improve hands-on skills and diagnostic capabilities [17]. Incorporating these innovative teaching methods can help students gain a better understanding of orthodontic principles and treatments. Research by Singh and Sharma examined the impact of patient-dentist communication on treatment satisfaction and outcomes [18]. This study suggests that dental curricula should emphasize communication skills to help future dentists manage patient expectations and concerns effectively, particularly in relation to orthodontic treatment.

Dental education plays a pivotal role in shaping the perspectives and practices of future dental professionals regarding orthodontic treatment. Understanding the motivations, attitudes, and awareness levels of dental students toward malocclusion and orthodontic interventions is crucial for addressing the evolving demands and expectations of patients. Research suggests that while esthetic concerns remain a primary driver for seeking orthodontic treatment, there is a growing recognition of its broader impact on psychosocial well-being and overall quality of life. Dental education programs should aim to incorporate discussions on the psychological benefits of orthodontic intervention, alongside traditional clinical considerations, to equip students with a comprehensive understanding of patient needs and preferences.

Furthermore, exploring the factors influencing patients' decision-making processes regarding orthodontic treatment can inform dental education strategies and treatment planning approaches. Key determinants such as treatment cost, duration, effectiveness, and provider expertise, as well as patient concerns regarding pain, discomfort, and inconvenience, should be addressed within dental curricula to prepare students for patient-centered care delivery. By acknowledging and addressing these factors, dental education programs can better prepare students to meet the diverse needs of patients seeking orthodontic treatment, ultimately enhancing patient satisfaction and treatment outcomes in clinical practice.

The study limitations are the following: A larger and more diverse sample would enhance the study's external validity and generalizability of results. The data collection relied solely on self-reported responses from participants via a standardized questionnaire, which may introduce response bias and inaccuracies. Addressing these limitations in future studies would strengthen the evidence base and contribute to more informed dental education and patient care practices.

## Conclusions

Subjects were aware that their teeth do affect their facial appearance and orthodontic treatment can enhance their profile. The subjects felt good about getting braces. Since all four years of BDS students are in the same age bracket (17-25), they all have the same understanding of what orthodontic treatment is and how to address malocclusion; therefore, there was no discernible difference in their knowledge of the issue or their attitude toward it. The present study concludes that dental students show optimal awareness about orthodontic diagnosis and treatment, but the inclusion of more clinical exposure to orthodontic treatment during the undergraduate course will encourage further interest in the subject.

## Appendices

Questionnaire:

· Age:

· Gender:

· Year of study:

· Have you received formal education in orthodontics during your dental studies?

*Yes       *No      *Don’t know

· Do you think healthy and well-arranged teeth are important for your appearance?

*Yes       *No      *Don’t know

· Are you satisfied with your dental appearance?

*Yes       *No      *Don’t know

· How do you feel about the appearance of your teeth?

*Yes       *No      *Don’t know

· Do you have any trouble while speaking or chewing or facial muscle pains caused by teeth arrangement?

*Yes       *No      *Don’t know

· Do you think you should have orthodontic treatment?

*Yes       *No      *Don’t know

· Would you agree readily to orthodontic treatment if a dentist or parent suggested it?

*Yes       *No      *Don’t know

· Do you think your smile could be better if your teeth were better aligned?

*Yes       *No      *Don’t know

· Have you found that other people have commented on the appearance of your teeth?

*Yes       *No      *Don’t know

· Have you found that other people have teased you on the appearance of your teeth?

*Yes       *No      *Don’t know

· Do you try to avoid smiling because of the appearance of your teeth?

*Yes       *No      *Don’t know

· Do you ever cover your mouth because of your teeth?

*Yes       *No      *Don’t know

· What kind of teeth do you have?

*Yes       *No      *Don’t know

· Are your teeth well aligned?

*Yes       *No      *Don’t know

· How much do you like the way your teeth look?

*Yes       *No      *Don’t know

· How much do your teeth affect the way your face looks?

*Yes       *No      *Don’t know

· Do you think your smile could be better if your teeth were better aligned?

*Yes       *No      *Don’t know
